# Ultra-high electrochemical catalytic activity of MXenes

**DOI:** 10.1038/srep32531

**Published:** 2016-09-08

**Authors:** Hui Pan

**Affiliations:** 1 Institute of Applied Physics and Materials Engineering, Faculty of Science and Technology, University of Macau, Macao SAR

## Abstract

Cheap and abundant electrocatalysts for hydrogen evolution reactions (HER) have been widely pursued for their practical application in hydrogen-energy technologies. In this work, I present systematical study of the hydrogen evolution reactions on MXenes (Mo_2_X and W_2_X, X = C and N) based on density-functional-theory calculations. I find that their HER performances strongly depend on the composition, hydrogen adsorption configurations, and surface functionalization. I show that W_2_C monolayer has the best HER activity with near-zero overpotential at high hydrogen density among all of considered pure MXenes, and hydrogenation can efficiently enhance its catalytic performance in a wide range of hydrogen density further, while oxidization makes its activity reduced significantly. I further show that near-zero overpotential for HER on Mo_2_X monolayers can be achieved by oxygen functionalization. My calculations predict that surface treatment, such as hydrogenation and oxidization, is critical to enhance the catalytic performance of MXenes. I expect that MXenes with HER activity comparable to Pt in a wide range of hydrogen density can be realized by tuning composition and functionalizing, and promotes their applications into hydrogen-energy technologies.

As an important energy carrier, hydrogen is clean, abundant, and renewable, and has been extensively investigated for its practical applications in green-energy technologies. Series of hydrogen-related technologies have been developed for their practical applications, such as hydrogen production and utilization in fuel cell^1^,^2^,^3^,^4^,^5^,^6^. Hydrogen evolution reaction (HER) is involved in the electrochemical reactions of both these technologies and determines the efficiencies of hydrogen production and utilization. The electrocatalyst used in these technologies plays a key role on the efficient HER reactions. Currently, noble metals, such as platinum and palladium, are most common electrocatalysts in electrolysis of water and fuel cell because of their high catalytic performance in HER^7^,^8^,^9^,^10^,^11^,^12^. However, their ultra-high cost and very-low abundance are detrimental to the commercialization of these technologies on large scale. Extensive efforts have been carried out to reduce the amounts of noble metals by alloying with cheap metals and tuning the composition of the catalysts^7^,^8^,^9^,^10^,^11^,^12^. Alternatively, novel electrocatalysts with low cost and rich abundance have been widely investigated to replace noble-metal catalysts^13^,^14^,^15^,^16^,^17^,^18^,^19^,^20^,^21^,^22^,^23^,^24^,^25^,^26^,^27^,^28^,^29^,^30^,^31^,^32^,^33^,^34^,^35^. Especially, two-dimensional (2D) monolayers as electrocatalysts, such as 2D transition-metal dichalcogenides monolayers (TMDs), have been attracting increasing interests because of their unique physical and chemical properties^20^,^21^,^22^,^23^,^24^,^25^,^26^,^27^,^28^,^29^,^30^,^31^,^32^,^33^,^34^,^35^. Experimental and theoretical studies showed that the electrocatalytic activity strongly depended on their structure, conductivity, edge states, defects, tensile strain, etc^20^,^21^,^22^,^23^,^24^,^25^,^26^,^27^,^28^,^29^,^30^,^31^,^32^,^33^,^34^,^35^. For example, the electrocatalytic activities of semiconducting TMDs in electrolysis of water were contributed to their metallic edges^25^,^29^. Further studies showed that the surfaces of metallic TMDs showed better HER performance than semiconducting counterparts^26^,^28^,^31^,^33^. However, most TMDs only showed electrocatalytic activity at low hydrogen coverage on surfaces or at edges because their conductivities were reduced or metallic TMDs changed to semiconducting ones as hydrogen coverage increases, which dramatically limits their practical applications^33^.

Recently, a new family of 2D monolayers, MXenes, were discovered^36^,^37^,^38^,^39^,^40^,^41^,^42^,^43^,^44^,^45^,^46^,^47^,^48^,^49^,^50^,^51^,^52^. The MXenes are transitional-metal carbides/nitrides monolayers and have a general formula, M_i+1_X_i_, where “M” is transition metal element, “X” is C or N, and i is a positive integer^36^. These monolayer with various thicknesses can be simply obtained by exfoliating layered ternary transition metal carbides/nitrides (M_i+1_AX_i_), where “A” is main group element (group IIIA and IVA)^36^. Most recently, Xu *et al.* reported the growth of large-scale high quality, superconducting 2D Mo_2_C monolayer by chemical vapour deposition^52^. Theoretical and experimental studies showed that most of MXenes are metallic. As learnt from the noble metals and MX_2_ monolayers/nanoribbons, it is well-known that the high conductivity of electrocatalyst is the prerequisite to the excellent HER activity. It is, therefore, that MXenes may find applications as electrocatalysts in hydrogen-related green energy technologies. To date, the study on the HER performances of MXenes has not been available. In this work, the electrocatalytic performance of MXenes for their applications as catalysts in HER is investigated based on the calculations of density-functional theory (DFT). It is predicted that the HER performances of MXenes strongly depend on their composition, surface treatment, hydrogen coverage, and hydrogen adsorption sites. It is found that pure and hydrogenated W_2_C monolayers are excellent in HER in a wide range of hydrogen density, while oxidization results in the significant reduction of its HER ability. It is shown that oxidized Mo_2_X monolayer is much better than pure and hydrogenated counterparts in HER. It is suggested that surface treatment is crucial to the applications of MXenes as electrocatalysts in HER.

## Results

### Geometric structure

**Figure 1:**
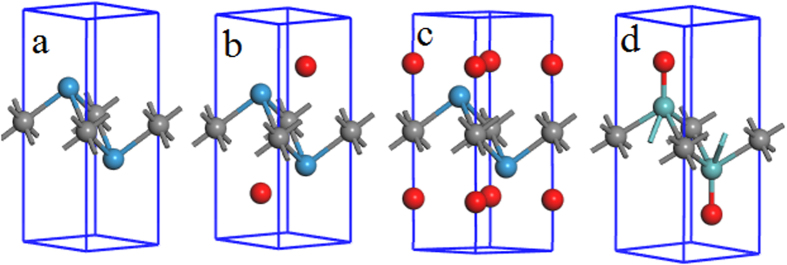
The representative structures of (**a**) pristine M_2_X and M_2_X with both sides covered by atoms (red) at different positions: (**b**) HC, (**c**) TX, and (**d**) TM. By removing red atoms from one side of the monolayer, one-side coverage is realized.

In the calculations, I focus on molybdenum- and tungsten-based MXenes, M_2_X (M = Mo and W; X = C and N). M_2_X monolayer is a three-atom-thick layer in a sequence of M1-X-M2 (Fig. 1a), and X-atom layer is enclaved in M-atom layers, leading to M_6_N octahedron. The unit cells of these MXenes are fully relaxed to obtain their lattice constants and study their electronic properties. It is noticed that the lattice constants of M_2_C MXenes are larger than those of M_2_N, and the lattice constants of Mo_2_X are larger than those of W_2_X (Table 1). The calculated total density of states (TDOSs) show that M_2_X monolayers are metallic (Supporting Data, S1), indicating their potential applications as electrocatalysts in HER.

### Hydrogen adsorption

**Figure 2:**
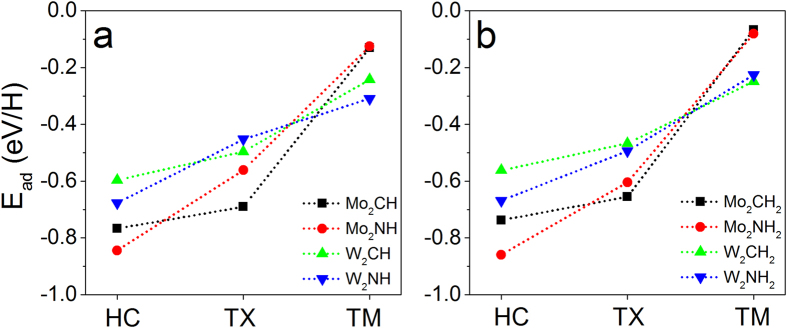
Calculated H-adsorption energy at different sites on M_2_X monolayers: (**a**) one-side H-coverage and (**b**) two-side H-coverage.

To investigate their HER abilities, the lattice parameters of hydrogen-covered MXenes need to be calculated. Generally, there are three possible sites for hydrogen atoms to be adsorbed on the monolayers, including top of hexagonal center (HC) (Fig. 1b), top of X atom (TX) (Fig. 1c), and top of M atom (TM) (Fig. 1d). To give a full understanding on the effect of hydrogen coverage on their HER abilities, two cases are considered, including one side and both sides of MXenes covered by hydrogen atoms. The unit cells of MXenes with various hydrogen coverages at different adsorption sites are fully relaxed to find the stable adsorption position and obtain lattice parameters. For simplicity, these MXenes with various hydrogen coverages at different adsorption sites are named as M_2_XH_m_-Ad, where m = 1 (H-coverage on one side of monolayer) and m = 2 (H-coverage on both sides of monolayer), and “Ad” is the adsorption site and can be HC, TX, and TM. The relaxed geometries show that the adsorption of hydrogen atoms on HC and TX of MXene unit cell has negligible effect on the lattice constants, while that on TM results in the lattice extension by 1~8% (Table 1). At the same time, the thicknesses of the monolayers are increased by ~5% when hydrogen atoms adsorb on HC and TX, but reduced by 10~20% for hydrogen adsorption on TM. As an indication of stable adsorption, the adsorption energy (E_ad_) is calculated as below:


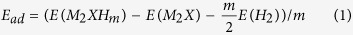


where E(M_2_XH_m_) and E(M_2_X) are the total energies of MXene unit cell with and without H atoms (*m*), and *E*(*H*_2_) is the energy of hydrogen molecule (H_2_). *m* is 1 for H-coverage on one side of monolayer or 2 for H-coverage on its both sides. Our calculations shows that the adsorption energies are negative at all of three possible sites (Fig. 2), indicating that all of the sites may be possible to host hydrogen atoms. It is found that HC is the most stable site to host hydrogen atom, followed by TX, and then by TM because the adsorption energy (negative) increases as the adsorption site changing from HC → TX → TM (Fig. 2). It is also found that the adsorption energy difference between two sites on W_2_C monolayer is the smallest among all of the considered MXenes. The variation of adsorption energy may affect the HER ability of MXenes.

### HER activity of pure MXenes

Basically, an advanced catalyst for the enhanced electrochemical hydrogen evolution reaction should reduce the HER reaction overpotential and consequently increase the HER efficiency, which can be quantified by the reaction Gibbs free energy of hydrogen adsorption (ΔG_H_)^9^,^10^,^11^,^53^. To investigate the hydrogen-coverage (H-coverage) dependent HER activity of pure MXene, a supercell with 2 × 2 × 1 unit cells is constructed based on the unit of MXene with one surface fully covered by hydrogen atoms at different adsorption sites (M_2_XH-Ad). Partial H-coverage is realized by removing H atom one by one from the H-covered surface of M_2_XH-Ad and the calculation on H-coverage-dependent HER performance is carried out accordingly. The H-coverage is defined as 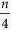 (n = 0~4). The H-coverage dependent ΔG_H_ can be calculated as below^10^,^11^,^31^,^53^:


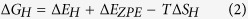


where Δ*E*_*H*_ is the hydrogen chemisorption energy. It can be differential chemisorption energy as calculated from:


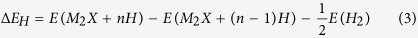


or, be average chemisorption energy as calculated from:


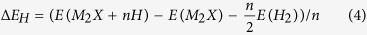


where *n* ( = 0~4) is the number of H atoms adsorbed on one side of a M_2_X monolayer. The H-coverage-dependent *ΔG*_*H*_ can be obtained by changing *n. E*(*M*_*2*_*X + nH*) and *E*(*M*_*2*_*X*) in Eqs (3) and (4) are the energies of monolayer supercell with variable hydrogen atoms (n) and pure M_2_X supercell, respectively. Δ*S*_*H*_ is the difference in entropy and T is room temperature. Δ*E*_*ZPE*_ is the difference in zero point energy between the adsorbed and the gas phase. Δ*E*_*ZPE*_ − *T*Δ*S*_*H*_ is about 0.24 eV. Therefore, Eq. (2) can be simplified to Δ*G*_*H*_ = Δ*E*_*H*_ + 0.24. According to the two methods (Eqs (3) and (4)) for the calculation of hydrogen chemisorption energy, the Gibbs free energies are defined as differential ΔG_H_ (d-ΔG_H_) and average ΔG_H_ (a-ΔG_H_), which can be used to express the HER activities in the individual and collective processes, respectively. The individual process describes that hydrogen is produced one by one, while the collective process shows that all of hydrogen atoms on the surface are simultaneously converted to molecules. In principle, an electrocatalyst with optimal HER performance should have a ΔG_H_ near 0 eV.

**Figure 3:**
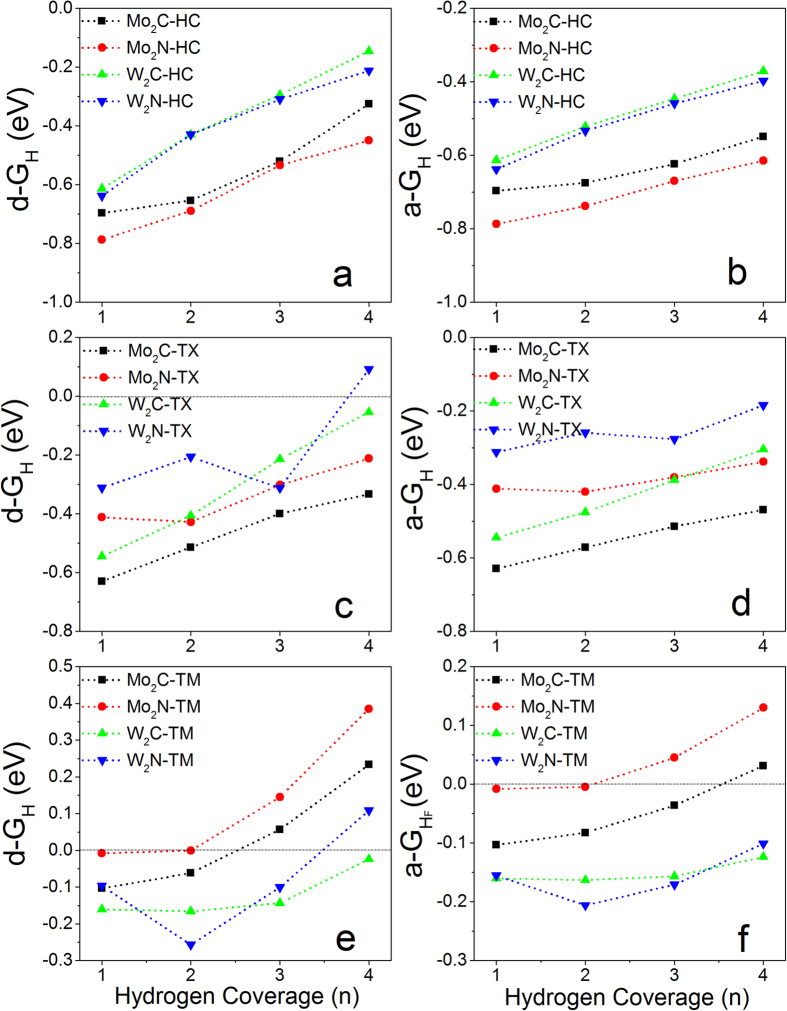
Calculated differential Gibbs free energy as a function of H-coverage on pure M_2_X monolayers (M_2_X-Ad) with H atoms adsorbed on: (**a**) HC, (**c**) TX, and (**e**) TM; Calculated average Gibbs free energy as a function of H-coverage on pure M_2_X monolayers with H atoms adsorbed on: (**b**) HC, (**d**) TX, and (**f**) TM.

The calculated ΔG_H_ shows that the HER activities of M_2_X monolayers strongly depend on the H-coverage and adsorption sites (Fig. 3). For M_2_X monolayers with H adsorbed on HC (M_2_X-HC), the calculated Gibbs free energies are negative and increase with the increment of H-coverage in both individual and collective processes (Fig. 3a,b). The W_2_X monolayers are better than Mo_2_X in the HER activity because of their relatively lower overpotentials (absolute values of ΔG_H_). Especially, the d-ΔG_H_ of W_2_C-HC is about −0.15 eV at full H-coverage (n = 4) (Fig. 3a), indicating good HER performance at high H-density in individual process. The calculated average Gibbs free energies (a-ΔG_H_) for M_2_X-HC are less than −0.3 eV (Fig. 3b). The collective processes are more difficult to take place than individual processes because the total energy in the collective process needs to multiply the number hydrogen atoms removed from the surface, which are far away from 0 eV (Fig. 3b).

If the hydrogen atoms adsorb on TX sites of M_2_X monolayers (M_2_X-TX), it is found that the HER activities are improved because of relatively lower overpotentials (Fig. 3c,d) at the same H-coverage compared to M_2_X-HC (Fig. 3a,b). In particular, the differential Gibbs free energies of W_2_C-TX and W_2_N-TX monolayers at full H-coverage (n = 4) are −0.05 and 0.09 eV (Fig. 3c), respectively, which are much near zero than those with H atoms adsorbed on HC sites (−0.14 eV for W_2_C and −0.21 eV for W_2_N, Fig. 3a). Similarly, the collective processes are difficult because their overpotentials are larger than those in individual processes (Fig. 3c,d).

Different from H atoms adsorbed on HC and TX, all of considered MXenes with H atoms adsorbed on TM show good HER activities at certain H-coverage because of near zero overpotentials in both individual and collective processes (Fig. 3e,f). For example, Mo_2_N monolayer (Mo_2_N-TM) shows the best HER performance at low H-coverage in both individual and collective processes due to near-zero d-ΔG_H_ (−0.008 eV for n = 1 and −0.001 eV for n = 2) (Fig. 3e) and a-ΔG_H_ (−0.008 eV for n = 1 and −0.004 eV for n = 2) (Fig. 3f). W_2_C monolayer (W_2_C-TM) still shows the best HER activity with d-ΔG_H_ = −0.02 eV at high H-coverage (n = 4) in individual process (Fig. 3e). Mo_2_C-TM is excellent for HER reactions at low hydrogen density in individual process (d-ΔG_H_ = −0.1 for n = 1 and −0.06 for n = 2) (Fig. 3e) and at high hydrogen density in collective process (a-ΔG_H_ = −0.04 eV for n = 3 and 0.03 eV for n = 4) (Fig. 3f). Clearly, the weak chemical-adsorption leads to the enhancement of HER activity (Fig. 3).

### HER activity of MXenes with one side hydrogenated

**Figure 4:**
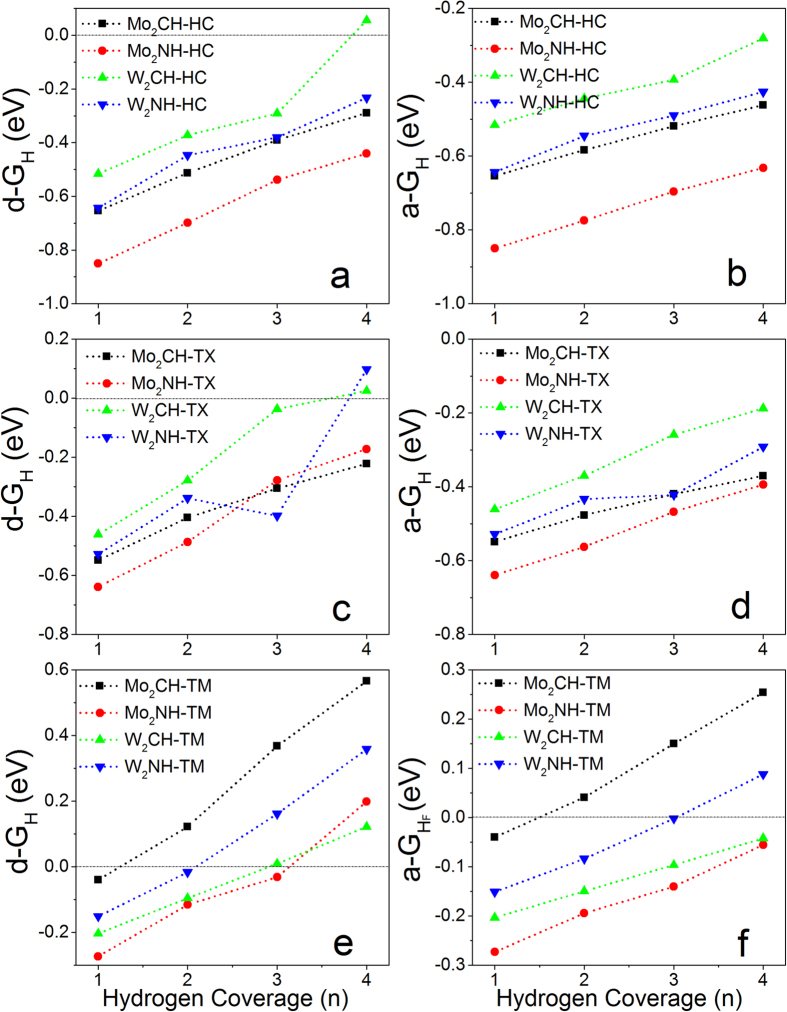
Calculated differential Gibbs free energy as a function of H-coverage on one-side hydrogenated M_2_X monolayers (M_2_XH-Ad) with H atoms adsorbed on: (**a**) HC, (**c**) TX, and (**e**) TM; Calculated average Gibbs free energy as a function of H-coverage on one-side hydrogenated M_2_X monolayers with H atoms adsorbed on: (**b**) HC, (**d**) TX, and (**f**) TM.

It had been reported that MXene can be easily functionalized by H, OH, and F, which affect their performance in energy storage^40^,^41^,^43^. To study the effect of hydrogenation on HER activity, a supercell with 2 × 2 × 1 unit cells is constructed based on the unit of MXene with both surfaces fully covered by hydrogen atoms at different adsorption sites (M_2_XH_2_-Ad). By removing H atom one by one from one of its surfaces, the effect of hydrogenation on the H-coverage dependent HER activity can be evaluated. To calculate the Gibbs free energies, it only needs to replace M_2_X in Eqs (3) and (4) by M_2_XH. Therefore, *E*(*M*_*2*_*XH + nH*) is the energy of monolayer with one side covered with variable hydrogen atoms (n) and another side fully covered by hydrogen atoms (M_2_XH-Ad-nH), and *E*(*M*_*2*_*XH*) is the energy of M_2_X monolayer with one side fully covered by hydrogen atoms (M_2_XH-Ad). The calculated overpotentials show that hydrogenation can efficiently improve the HER activities of M_2_X monolayers (Fig. 4). For M_2_X monolayer with hydrogenation at HC sites on one of its surfaces (M_2_XH-HC), the HER activities are improved because of the reduced overpotentials in individual processes (Fig. 4a). In particular, the d-ΔG_H_ of W_2_C monolayer is about 0.05 eV at full H-coverage (n = 4), which just satisfies the basic requirement for an efficient electrocatalyst with ΔG_H_ = 0 eV. Although the average Gibbs free energies (a-ΔG_H_ < 0) for M_2_XH-HC in collective processes are increased (Fig. 4b), they are still less than −0.2 eV, indicating that the collective processes are difficult to take place.

Interestingly, the hydrogenation on one side of W_2_C monolayer with H atoms at TX sites (W_2_CH-TX) can efficiently improve its catalytic performance at high H-density (Fig. 4c). The calculated differential Gibbs free energies of W_2_CH-TX are close to zero at high H-coverage (−0.04 and 0.02 eV for n = 3 and 4), indicating excellent HER performance. The calculated average Gibbs free energies (negative) (Fig. 4d) are less than differential ones (Fig. 4c), indicating that collective process is also difficult to happen.

Hydrogenation dramatically affect the HER performances of M_2_XH-TM monolayers in both individual and collective processes (Fig. 4e,f). It is found that all of M_2_XH-TM monolayers show excellent HER activities at certain H-coverage in both processes. The calculated d-ΔG_H_ and a-ΔG_H_ on Mo_2_CH-TM are −0.04 eV at n = 1. The Gibbs free energies in individual processes are −0.03 eV for Mo_2_NH-TM and −0.02 eV for W_2_NH-TM at n = 3, respectively. Although the HER activity of W_2_CH-TM is reduced at n = 4 (d-ΔG_H_ = 0.12 eV), it is strongly improved at n = 3 (d-ΔG_H_ = −0.01 eV) in individual process (Fig. 4e). Importantly, the calculated a-ΔG_H_ for Mo_2_NH-TM and W_2_CH-TM are about −0.04 eV at full H-coverage (n = 4) and that for W_2_NH-TM is about −0.02 eV at n = 3 (Fig. 4f), indicating the collective processes can take place. Compared to pure MXenes (Fig. 3), it is found that hydrogenation can efficiently enhance their HER performance, especially in the collective processes.

### HER activity of oxidized MXenes

**Figure 5:**
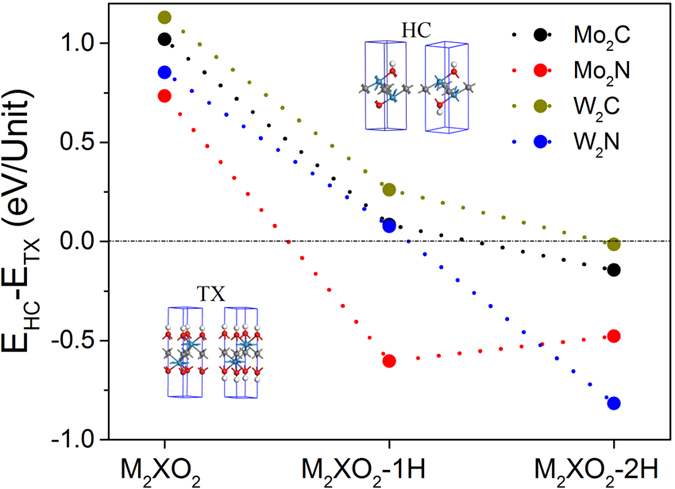
Calculate energy differences of M_2_X monolayers with oxidization at different sites and H-adsorption. The insets show the oxidized M_2_X monolayers with H atoms at HC and TX sites, respectively.

Beside hydrogenation, the MXenes are also easily oxidized. In the section, I focus on the effect of oxygen-functionalization on their HER activities. The MXenes with two sides oxidized are named as M_2_XO_2_ (M = Mo and W, and X = C and N). There are three possible sites for oxygen to cover the surfaces M_2_X monolayers (Fig. 1b–d). The unit cells of M_2_XO_2_-Ad monolayers (Ad = HC, TX, and TM) are fully relaxed to obtain the stable adsorption site. It is found that M_2_XO_2_-HC and M_2_XO_2_-TX are more stable than M_2_XO_2_-TM because their energies are low by ~3–4 eV/unit, and M_2_XO_2_-TX is more stable than M_2_XO_2_-HC by an energy of 0.7–1.2 eV/unit (Fig. 5). To investigate their applications in HER reactions, both M_2_XO_2_-HC and M_2_XO_2_-TX are studied because the adsorption of hydrogen may lead to phase transition. The unit cells of M_2_XO_2_ monolayers with H atoms adsorbed on the tops of oxygen atoms (M_2_XO_2_-mH-Ad, m = 1 and 2, Ad = HC and TX) are optimized (insets in Fig. 5). Our calculations show that the energy differences between M_2_XO_2_-1H-TX and M_2_XO_2_-1H-HC was reduced to 0.05~0.26 eV when one side is fully covered by hydrogen atoms, except Mo_2_NO_2_-1H (Fig. 5). Mo_2_NO_2_-1H-HC is more stable than Mo_2_NO_2_-1H-TX by an energy of 0.6 eV (Fig. 5), indicating phase transition from Mo_2_NO_2_-TX to Mo_2_NO_2_-HC. When both sides of M_2_XO_2_ monolayers are covered by hydrogen atoms (M_2_XO_2_-2H), phase transitions take place in all systems because M_2_XO_2_-2H-HC is more stable than M_2_XO_2_-2H-TX by an energy of 0.02~0.82 eV (Fig. 5). The calculated lattice constants of M_2_XO_2_-Ad and M_2_XO_2_-mH-Ad are larger than those of M_2_X (Supporting data, Table T1). Because of possible phase transition during hydrogen evolution reaction, both adsorption sites (HC and TX) are considered when calculating Gibbs free energies.

**Figure 6:**
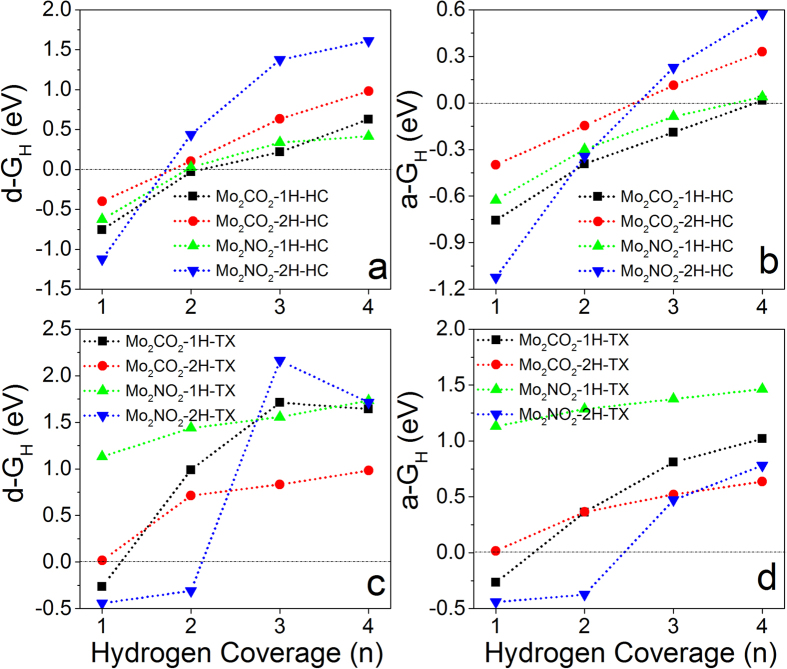
Calculated overpotentials as a function of H-coverage on oxidized Mo_2_X monolayers: (**a**) differential Gibbs free energy for H atoms adsorbed on HC sites, (**b**) average Gibbs free energy for H atoms adsorbed on HC sites, (**c**) differential Gibbs free energy for H atoms adsorbed on TX sites, (**b**) average Gibbs free energy for H atoms adsorbed on TX sites.

Similarly, a supercell with 2 × 2 × 1 unit cells is constructed based on the unit of M_2_XO_2_-mH-Ad (m = 1 and 2, Ad = HC and TX). By removing H atom one by one from a H-covered surface, the H-coverage dependent HER activity can be evaluated. The DFT-calculated Gibbs free energies show that Mo_2_X monolayers with oxidization at HC sizes are better than those at TX sites in HER activities (Fig. 6). Mo_2_XO_2_-1H-HC (X = C and N) shows the best HER performance at n = 2 (d-ΔG_H_ = −0.03~0.03 eV), and the HER activities of Mo_2_XO_2_-2H-HC are better if 1 < n < 2 where the d-ΔG_H_ cross the reference line (0 eV) in individual processes (Fig. 6a). Importantly, the a-ΔG_H_ of Mo_2_XO_2_-1H-HC (X = C and N) at full H-coverage (n = 4) are near-zero (0.01~0.04 eV, Fig. 6b), indicating the collective processes can take place. It is expected that the collective HER process on Mo_2_XO_2_-1H-HC is dominant at high H-density, and the individual processes may take over the role when H-density is low. Similarly, the collective processes of Mo_2_XO_2_-2H-HC can take place within the H-coverage from 2 to 3 (Fig. 6b). For Mo_2_X monolayers with oxidization at TX, only Mo_2_CO_2_-2H-TX shows high HER activity at low H-coverage (n = 1, ΔG_H_ = 0.02 eV) (Fig. 6c,d). Although the oxidization results in the improvement of the HER performances of W_2_CO_2_ monolayers with O atoms at HC sites at low H-coverage, but their HER activities at high H-coverage are greatly reduced (Supporting data, S-2a,b). W_2_CO_2_ monolayers with O atoms at TX sites shows poor HER performances (S-2c,d). Our calculations also show that W_2_NO_2_ monolayers with partial H-coverage (0 < n < 4) are unstable and their Gibbs free energies are not calculated.

## Discussion

By systematically analysing the HER activities of pure and functionalized M_2_X monolayers with various H-adsorption configurations, it is found that the HER activity of MXene strongly depends on its composition, hydrogen adsorption configuration, and surface functionalization. Generally, the less stable adsorption, the better HER activity. It is found that pure and hydrogen-functionalized W_2_C monolayers shows the best HER performance at high H-coverage among all of considered systems, and is comparable to Pt because of near-zero overpotential. The overpotentials of pure W_2_C monolayer at full H-coverage in individual process decreases from 0.15 eV to 0.05 eV, then to 0.02 eV as the H-adsorption site changing from HC → TX → TM (Fig. 3a,c,e). The hydrogen functionalization can efficiently improve the HER activity of W_2_C monolayer. Almost zero Gibbs free energies at high H-coverage in individual processes can be achieved on W_2_CH regardless of H-adsorption sites (Fig. 4a,c,e). Importantly, both differential and average Gibbs free energies of W_2_C-TM and W_2_CH-TM are within a range of −0.2 to 0.1 eV in the whole H-coverage (n = 1~4) (Figs 3e,f and 4e,f), indicating its excellent performance in hydrogen evolution reaction. Their HER activities are much better than MX_2_ monolayers and nanoribbons when considering the H-coverage density and oeverpotentials, where 2D MX_2_ only showed high activities at certain H-density^23^,^26^,^29^,^30^,^31^,^32^,^33^,^34^,^35^,^54^,^55^ (Supporting data, Table S-2). A recent work on V_2_CO_2_ was noticed during revision, which showed that pure monolayer is worse on HER, while metal-decoration on its surface could improve its HER performance at certain H-coverage^56^. My calculations predict that pure W_2_C, W_2_CH and Mo_2_XO_2_ (X = C and N) monolayers show better HER performances with near-zero Gibbs free energies in wide range of H-coverages than Ni-V_2_CO_2_ (Table S-2). Although H atoms prefer to occupy HC or TX sites on W_2_C monolayer (Fig. 2), the H adsorption on TM is also exothermic and the energy is only higher than those on HC and TX by 0.20~0.35 eV/unit. In experiments, H atoms may first approach to TM sites because metal layers are outmost, and then HER reactions occurs accordingly. Interestingly, the oxidized W_2_C monolayers show better HER activities with near-zero Gibbs free energy at low H-coverage, but worse at high H-coverage (S-2a,b). It is, therefore, suggested that acid solution is required in HER reactions to avoid oxidization and keep the HER performance of W_2_C monolayer. Different from oxidized W_2_C monolayers, the oxygen functionalized Mo_2_X monolayers shows excellent HER catalytic activities at medium H-coverage (n = 2) in individual processes (Fig. 6a) and at high H-coverage in collective processes (Fig. 6b). Especially, the average Gibbs free energies of Mo_2_XO_2_-1H-HC at full H-coverage (n = 4) in collective processes are 0.02~0.04 eV (Fig. 6b). Compared with pure and H-functionalized Mo_2_X monolayers (Figs 3 and 4), it is found that oxidization can dramatically improve their HER activities at full H-coverage in collective processes (Fig. 6b), but their HER performances at low H-coverage are greatly reduced.

## Conclusions

I carries out first-principles calculations to investigate the catalytic activities of MXenes for applications into HER. It is found that pure and hydrogenated W_2_C monolayers are better than other MXenes without oxygen functionalization and show the best HER performances at high H-density in HER reactions because of near-zero overpotential. In particular, excellent HER performance in the whole H-coverage from zero to full can be achieved on W_2_C monolayers with H atoms adsorbed on the top of W atoms, which can be realized by controlling experimental conditions. However, the optimal HER activity of W_2_C monolayer is degraded if it is oxidized or functionalized by oxygen atoms, especially at high H-coverage. It is further shown that the oxidization can dramatically improve the HER activities of Mo_2_X monolayers. The oxidized Mo_2_X monolayers exhibit best HER performances in individual processes at medium H-coverage and in collective processes at high H-coverage due to near-zero Gibbs free energies. For practical applications in experiments, I suggest that oxidization should be avoided to keep the advanced HER activity of W_2_C monolayer used as electrocatalyst, while Mo_2_X monolayers may need to be functionalized by oxygen to enhance their activities. The MXenes with activity comparable to novel metals may be obtained by tuning compositions and functionalizing, and find applications into HER reactions.

## Methods

The first-principles calculations are carried out to investigate the hydrogen evolution reaction of MXenes. The calculations are based on the density functional theory (DFT)^57^ and the Perdew-Burke-Eznerhof generalized gradient approximation (PBE-GGA)^58^. The projector augmented wave (PAW) scheme^59^,^60^ as incorporated in the Vienna ab initio simulation package (VASP)^61^ is used in the study. The Monkhorst and Pack scheme of k-point sampling is used for integration over the first Brillouin zone^62^. A 15 × 15 × 1 grid for k-point sampling for geometry optimization of unit cells, and an energy cut-off of 500 eV are consistently used in our calculations. A sufficiently large supercell is used so that the monolayers in neighbouring cells in the vertical direction are separated by a vacuum region of at least 20 Å. Dipole correction is not included. Good convergence is obtained with these parameters and the total energy was converged to 2.0 × 10^−5^ eV/atom. The error bar (or uncertainty) of the DFT calculation is less than 5 meV.

## Additional Information

**How to cite this article**: Pan, H. Ultra-high electrochemical catalytic activity of MXenes. *Sci. Rep.* **6**, 32531; doi: 10.1038/srep32531 (2016).

## Supplementary Material

Supplementary Information

## Figures and Tables

**Table 1:** Lattice parameters of pure and hydrogenated MXene monolayers: M_2_X (M = Mo and W; X = C and N).

	a (Å)	c (Å)	X-M (Å)	H-M (Å)
Mo_2_C	2.978	2.368	2.088	
Mo_2_CH-HC	2.928	2.533	2.139/2.082	1.981
Mo_2_CH-TX	2.971	2.405	2.120/2.059	2.059
Mo_2_CH-TM	3.085	2.199	2.086/2.100	1.712
Mo_2_CH_2_-HC	2.960	2.498	2.112	2..002
Mo_2_CH_2_-TX	2.951	2.458	2.101	2.058
Mo_2_CH_2_-TM	3.063	2.300	2.109	1.716
Mo_2_N	2.798	2.799	2.137	
Mo_2_NH-HC	2.791	2.854	2.170/2.135	1.982
Mo_2_NH-TX	2.804	2.831	2.168/2.133	1.993
Mo_2_NH-TM	2.831	2.759	2.160/2.117	1.696
Mo_2_NH_2_-HC	2.791	2.876	2.159	1.978
Mo_2_NH_2_-TX	2.797	2.916	2.176	1.992
Mo_2_NH_2_-TM	3.103	2.168	2.095	1.705
W_2_C	2.874	2.657	2.125	
W_2_CH-HC	2.885	2.668	2.149/2.119	1.994
W_2_CH-TX	2.917	2.571	2.133/2.105	2.039
W_2_CH-TM	3.027	2.337	2.102/2.103	1.714
W_2_CH_2_-HC	2.909	2.654	2.141	2.003
W_2_CH_2_-TX	2.941	2.526	2.116	2.068
W_2_CH_2_-TM	3.048	2.368	2.119	1.717
W_2_N	2.787	2.897	2.165	
W_2_NH-HC	2.779	2.949	2.192/2.166	1.995
W_2_NH-TX	2.790	2.945	2.192/2.173	1.992
W_2_NH-TM	2.815	2.882	2.201/2.144	1.696
W_2_NH_2_-HC	2.778	2.982	2.189	2.000
W_2_NH_2_-TX	2.790	3.000	2.200	1.992
W_2_NH_2_-TM	2.846	2.856	2.177	1.701

a is lattice constant, c is the thickness of the monolayer in vertical direction, X-M is the bond length, and H-M is the hydrogen-metal distance.
