# Supplementary material for: Ultra-high electrochemical catalytic activity of MXenes
Source: Sci Rep. 2016 Sep 8;6:32531. doi: 10.1038/srep32531 (PMC5015052; doi:10.1038/srep32531)
Supplement: Supplementary Information [file srep32531-s1.doc]

Supporting Data

Ultra-high electrochemical catalytic activity of MXenes

Hui Pan*

Institute of Applied Physics and Materials Engineering, Faculty of Science and Technology, University of Macau, Macau SAR, China

* H. Pan ([huipan@umac.mo](mailto:huipan@umac.mo)); Tel: (853)83794427; Fax: (853)28838314


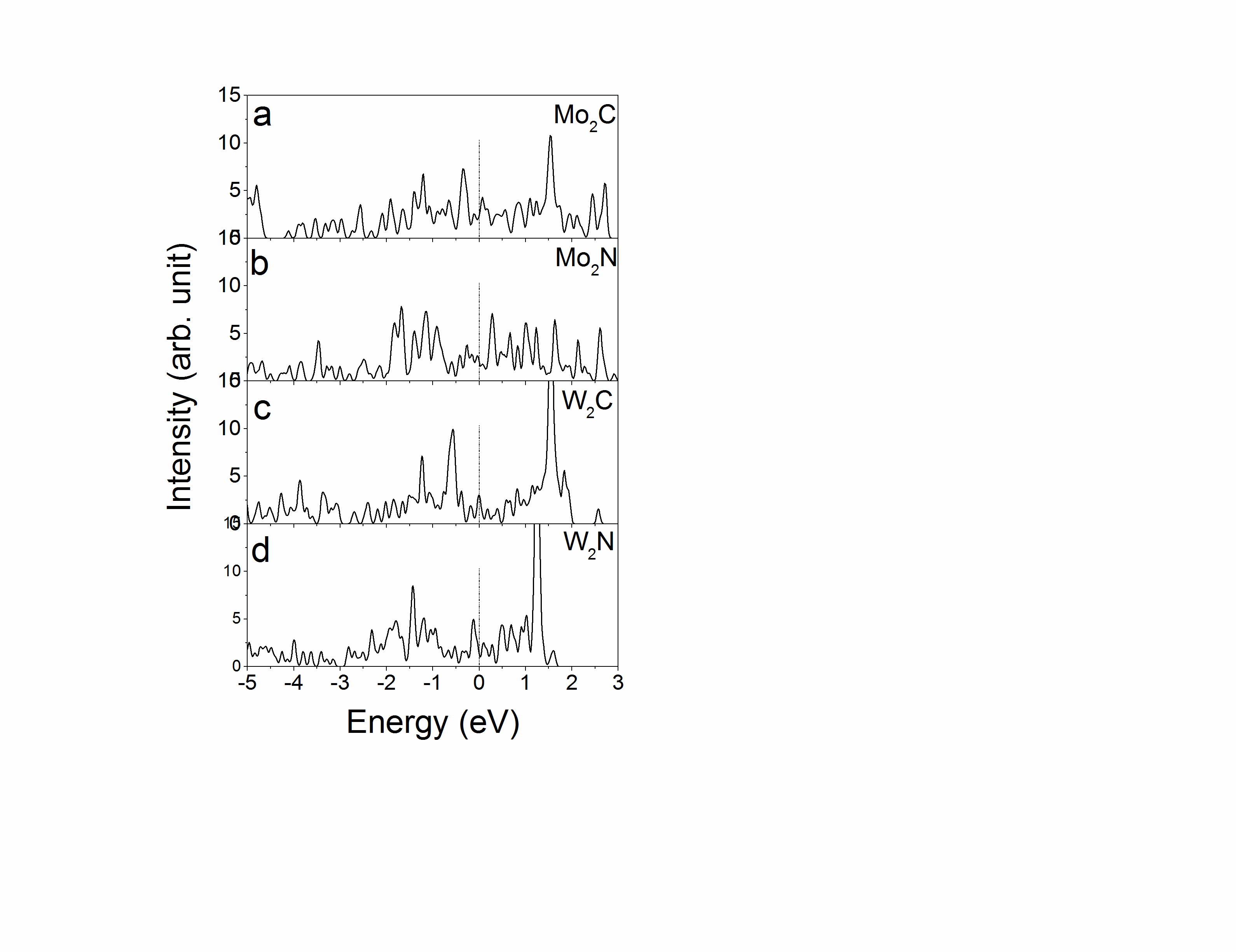


S1, Calculated total density of states: (a) Mo_2_C, (b) Mo_2_N, (c) W_2_C, and (5) W_2_N.


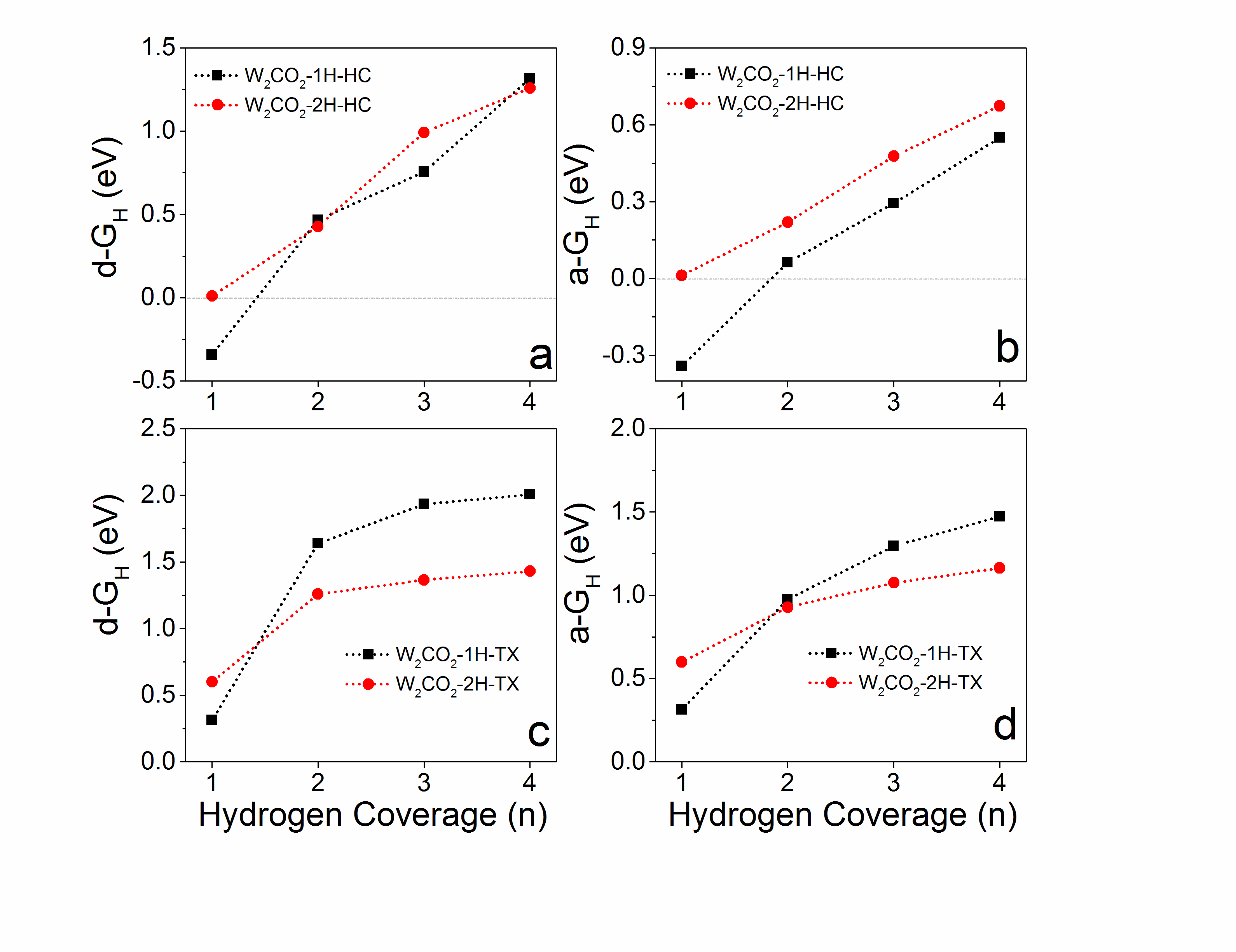


S-2, Calculated overpotentials as a function of H-coverage on oxidized W_2_C monolayers: (a) differential free Gibbs energy for H atoms adsorbed on HC sites, (b) average free Gibbs energy for H atoms adsorbed on HC sites, (c) differential free Gibbs energy for H atoms adsorbed on TX sites, (b) average free Gibbs energy for H atoms adsorbed on TX sites.

Table S-1, Lattice parameters of oxidized MXene monolayers with and with hydrogenation at HC or TX: M_2_XO_2_ (M = Mo and W; X = C and N). a is lattice constant, c is the thickness of the monolayer in vertical direction, and X-M is the bond length. O-M and H-O are the distances between O and metal/hydrogen.

|  | | a (Å) | c (Å) | X-M (Å) | O-M (Å) | H-O (Å) |
| --- | --- | --- | --- | --- | --- | --- |
| Mo_2_CO_2_ | HC | 3.096 | 2.234 | 2.108 | 2.107 | ---- |
|  | TX | 2.880 | 2.743 | 2.156 | 2.061 | ---- |
|  | 1H-HC | 3.154 | 2.111 | 2.036/2.181 | 2.325/2.110 | 0.973 |
|  | 1H-TX | 2.910 | 2.700 | 2.137/2.174 | 2.179/2.068 | 0.992 |
|  | 2H-HC | 3.286 | 1.894 | 2.120 | 2.304 | 0.974 |
|  | 2H-TX | 3.000 | 2.426 | 2.115 | 2.259 | 0.982 |
| Mo_2_NO_2_ | HC | 3.163 | 2.090 | 2.105 | 2.098 | ---- |
|  | TX | 2.871 | 2.820 | 2.176 | 2.041 | ---- |
|  | 1H-HC | 3.227 | 1.992 | 1.991/2.265 | 2.302/2.097 | 0.971 |
|  | 1H-TX | 2.874 | 2.772 | 2.155/2.169 | 2.218/2.020 | 0.996 |
|  | 2H-HC | 2.833 | 2.886 | 2.182 | 2.174 | 0.981 |
|  | 2H-TX | 3.031 | 2.317 | 2.098 | 2.228 | 0.980 |
| W_2_CO_2_ | HC | 3.118 | 2.211 | 2.112 | 2.128 | ---- |
|  | TX | 2.884 | 2.795 | 2.175 | 2.073 | ---- |
|  | 1H-HC | 3.170 | 2.105 | 2.047/2.182 | 2.342/2/127 | 0.978 |
|  | 1H-TX | 2.915 | 2.735 | 2.150/2.188 | 2.189/2.081 | 1.001 |
|  | 2H-HC | 3.318 | 1.866 | 2.130 | 2.323 | 0.978 |
|  | 2H-TX | 2.971 | 2.528 | 2.130 | 2.252 | 0.989 |
| W_2_NO_2_ | HC | 3.195 | 2.067 | 2.115 | 2.118 | ---- |
|  | TX | 2.882 | 2.860 | 2.194 | 2.056 | ---- |
|  | 1H-HC | 2.821 | 2.991 | 2.165/2.259 | 2.201/2.071 | 0.985 |
|  | 1H-TX | 2.861 | 2.872 | 2.209/2.169 | 2.239/2.060 | 1.003 |
|  | 2H-HC | 2.818 | 2.982 | 2.207 | 2.190 | 0.987 |
|  | 2H-TX | 3.019 | 2.373 | 2.108 | 2.295 | 0.987 |

**Table S-2, Calculated optimal Gibbs free energy on MX_2_ monolayers from literatures. BP – Basal Plane; P – Pure; I – Individual process; A – Average process**

| Materials | Phase | Catalytic sites | Sulfur coverage | Hydrogen coverage | Doping density | ∆G_H_ (eV) | I or A | Ref | remarks |
| --- | --- | --- | --- | --- | --- | --- | --- | --- | --- |
| MoS_2_ | 1T’ | BP |  | 1/16 | P | 0.13 | A | 1 | ∆G_H_=∆E_H_+0.29 |
|  | 1T’ | BP |  | 2/16 | P | 0.16 | A | 1 |  |
|  | 1T’ | BP |  | 4/16 | P | 0.18 | A | 1 |  |
|  | 1T’ | BP |  | 8/16 | P | 0.255 | A | 1 |  |
|  | 2H | Mo-edge | 4/8 | 2/4 | P | 0.06 | I | 2 |  |
|  | 2H | Mo-edge | Full | 1/8 | 4/4 Ni | 0.15 | I | 2 |  |
|  | 2H | Mo-edge | 4/8 | 2/4 | P | 0.08 | I | 3 | ∆G_H_=∆E_H_+0.29 |
|  | 2H | S-edge | 6/8 | 1/6 | 4/4 V | 0.08 | I & A | 4 |  |
|  | 2H | S-edge | Full | 3/8 | 4/4 Ta | 0.06 | I & A | 4 |  |
|  | 2H | S-edge | 4/8 | 3/4 | 4/4 Mn | 0.05 | I & A | 4 |  |
|  | 2H | S-edge | 4/8 | 1/4 | 4/4 Fe | 0.04 | I & A | 4 |  |
|  | 2H | S-edge | 4/8 | 2/4 | 4/4 Ru | 0.01 | I & A | 4 |  |
|  | 2H | S-edge | 4/8 | 1/4 | 4/4 Co | 0.01 | I & A | 4 |  |
|  | 2H | S-edge | 4/8 | 1/4 | 4/4 Rh | 0.01 | I & A | 4 |  |
| MoSe_2_ | 2H | Se-edge | Full | 4/8 | P | -0.05 | I | 2 |  |
|  | 2H | Mo-edge | 4/8 | 1/4 | P | 0.02 | I | 2 |  |
|  | 2H | Mo-edge | 6/8 | 1/6 | P | -0.04 | I | 2 |  |
| WS_2_ | 1T | BP |  | 1/16 | P | 0.28 ~ -0.16 | I | 5 | Tension from 0.0% ~ 4.0% & ∆G_H_ = ∆E_H_ + 0.28 |
|  | 2H | S-edge | Full | 4/8 | P | -0.06 | I | 2 |  |
|  | 2H | W-edge | 4/8 | 1/4 | P | -0.04 | I | 2 |  |
| WSe_2_ | 2H | Se-edge | Full | 1/16 | P | -0.05 | I | 2 |  |
|  | 2H | W-edge | 4/8 | 1/4 | P | 0.17 | I | 2 |  |
| VS_2_ | 2H | BP |  | 1/12 | P | 0.007 | I | 6 |  |
|  | 2H | BP |  | 1/6 | P | 0.051 | I | 6 |  |
|  | 2H | BP |  | 9/9 | 1/9 W | 0.09 | I | 7 |  |
|  | 2H | BP |  | 2/9 | 1/9 Ti | -0.05 | I | 7 |  |
|  | 2H | S-edge | Full | 5/8 | P | 0.006 | A | 8 |  |
|  | 2H | S-edge | Full | 3/8 ~ 8/8 | P | -0.12 ~ 0.10 | A | 8 |  |
|  | 2H | BP |  | 2, 3/16 | P | -0.04 & -0.03 | I | 9 | Tension 2% |
|  | 2H | BP |  | 4, 5/16 | P | -0.02 & 0.02 | I | 9 | Tension 6% |
|  | 2H | BP |  | 6, 7, 9/16 | P | 0.02, 0.04 & -0.03 | I | 9 | Tension 10% |
|  | 2H | BP |  | 5/16 | P | -0.007 | A | 9 | Tension 2% |
|  | 2H | BP |  | 8/16 ~10/16 | P | -0.005 ~ 0.02 | A | 9 | Tension 6% |
|  | 2H | BP |  | 14/16 | P | -0.016 | A | 9 | Tension 10% |
|  | 1T | BP |  | 1/16 | P | 0.05 | I | 10 |  |
| V_2_CO_2_ |  | BP |  | 1/8 | 1/8 Ni-absorbed | -0.15 | I | 11 |  |
|  |  | BP |  | 1/6 | 1/6 Ni-absorbed | -0.08 | I | 11 |  |
|  |  | BP |  | 1/4 | 1/4 Ni-absorbed | -0.01 | I | 11 |  |

References:

1. S.S. Chou, N. Sai, P. Lu, E.N. Coker, S. Liu, K. Artyushkova, T.S. Luk, B. Kaehr. C.J. Brinker. Understanding catalysis in a multiphasic two-dimensional transition metal dichalcogenide. Nat. Commun. 2015, 6: 8311.
2. C. Tsai, K. Chan, F.A. Pedersen, J.K. Nørskov. Active edge sites in MoSe_2_ and WSe_2_ catalysts for the hydrogen evolution reaction: a density functional study. Phys. Chem. Chem. Phys. 2014, 16: 13156.
3. B. Hinnemann, P.G. Moses, J. Bonde, K.P. Jørgensen, J.H. Nielsen, S. Horch, I.B. Chorkendorff, J.K. Nørskov. Biomimetic hydrogen evolution: MoS_2_ nanoparticles as catalyst for hydrogen evolution. J. Am. Chem. Soc., 2005, 127: 5308.
4. C. Tsai, K Chan, J.K. Nørskov, F.A. Pedersen. Rational design of MoS_2_ catalysts: tuning the structure and activity via transition metal doping. Catal. Sci. Technol. 2015, 5: 246.
5. D. Voiry, H. Yamaguchi, J. Li, R. Silva, D.C.B. Alves, T. Fujita, M. Chen, T. Asefa, V.B. Shenoy, G. Eda, M. Chhowalla. Enhanced catalytic activity in strained chemically exfoliated WS_2_ nanosheets for hydrogen evolution. 2013, 12: 850.
6. H. Pan, Metal dichalcogenides monolayers: novel catalysts for electrochemical hydrogen production. Sci. Rep. 2014, 4: 5348.
7. Y.J. Qu, H. Pan, C.T. Kwok, Z.S. Wang. Effect of doping on hydrogen evolution reaction of vanadium disulfide monolayer. Nanoscale Res. Lett. 2015, 10: 480.
8. Y.J. Qu, H. Pan, C.T. Kwok, Z.S. Wang. A first-principles study on the hydrogen evolution reaction of VS_2_ nanoribbons. Phys. Chem. Chem. Phys. 2015, 17: 24820.
9. H. Pan. Tension-enhanced hydrogen evolution reaction on vanadium disulfide monolayer. Nanoscale Res. Lett. **11, 113** (2016).
10. X. L. Fan, S. Y. Wang, Y. R. An, and W. M. Lau, Catalytic activity of MS_2_ monolayer for electrochemical hydrogen evolution. J. Phys. Chem. C 2016, 120: 1623.
11. C. Y. Ling, L. Shi, Y. X. Ouyang, Q. Chen, and J. L. Wang, Transition metal-promoted V_2_CO_2_ (MXenes): A new and highly active catalyst for hydrogen evolution reaction. Adv. Sci. 2016, 1600180.
